# Anti-*Leishmania amazonensis* Activity, Cytotoxic Features, and Chemical Profile of *Allium sativum* (Garlic) Essential Oil

**DOI:** 10.3390/tropicalmed8070375

**Published:** 2023-07-21

**Authors:** Andreza R. Garcia, Mariana M. B. Amorim, Ana Claudia F. Amaral, Jefferson D. da Cruz, Alane B. Vermelho, Dirlei Nico, Igor A. Rodrigues

**Affiliations:** 1 Programa de Pós-graduação em Ciências Farmacêuticas, Faculdade de Farmácia, Universidade Federal do Rio de Janeiro, Rio de Janeiro 21941-902, Brazil; deh.raposo@yahoo.com.br; 2 Instituto Municipal de Vigilância Sanitária, Vigilância de Zoonoses e de Inspeção Agropecuária, Rio de Janeiro 22290-240, Brazil; marimbarros@gmail.com; 3 Departamento de Produtos Naturais, Farmanguinhos Fiocruz, Manguinhos, Rio de Janeiro 21041-250, Brazil; acamaral@fiocruz.br (A.C.F.A.);; 4 Departamento de Microbiologia Geral, Instituto de Microbiologia Paulo de Góes, Universidade Federal do Rio de Janeiro, Rio de Janeiro 21941-902, Brazil; abvermelho@micro.ufrj.br; 5 Departamento de Produtos Naturais e Alimentos, Faculdade de Farmácia, Universidade Federal do Rio de Janeiro, Rio de Janeiro 21941-902, Brazil

**Keywords:** tegumentary leishmaniasis, antileishmanial activity, organosulfur compounds, cytotoxicity, computational analysis

## Abstract

Human tegumentary leishmaniasis (HTL) is a serious tropical disease caused by *Leishmania amazonensis*. Developing new leishmanicidal agents can help overcome current treatment challenges, such as drug resistance and toxicity. Essential oils are a source of lipophilic substances with diverse therapeutic properties. This study aimed to determine the anti-*L. amazonensis* activity, cytotoxicity, and chemical profile of *Allium sativum* essential oil (ASEO). The effect of ASEO on parasite and mammalian cells viability was evaluated using resazurin and MTT assays, respectively. The oil’s effect against intracellular amastigotes was also determined. Transmission electron microscopy was used to assess the ultrastructural changes induced by ASEO. In addition, the chemical constituents of ASEO were identified by gas chromatography-mass spectrometry (GC-MS). The cytotoxic potential was evaluated in vitro and in silico. The oil displayed IC_50_ of 1.76, 3.46, and 3.77 µg/mL against promastigotes, axenic, and intracellular amastigotes, respectively. Photomicrographs of treated parasites showed plasma membrane disruption, increased lipid bodies, and autophagic-like structures. ASEO chemical profiling revealed 1,2,4,6-tetrathiepane (24.84%) and diallyl disulfide (16.75%) as major components. Computational pharmacokinetics and toxicological analysis of ASEO’s major components demonstrated good oral bioavailability and better toxicological endpoints than the reference drugs. Altogether, the results suggest that ASEO could be an alternative drug candidate against HTL.

## 1. Introduction

Leishmaniasis is considered a Neglected Tropical Disease (NTD) due to the low investment in research and development of effective vaccines and medicines [1]. The World Health Organization (WHO) estimates that approximately one billion individuals reside in areas with a high risk of infection [2]. The disease is caused by parasites from the *Leishmania* genus (Trypanosomatidae) that are responsible for a wide range of clinical manifestations [3]. The tegumentary form, also known as human tegumentary leishmaniasis (HTL), can be classified into three main clinical forms: cutaneous leishmaniasis (CL), mucocutaneous leishmaniasis (MCL), and diffuse cutaneous leishmaniasis (DCL) [4]. HTL is the most prevalent form of leishmaniasis worldwide, with over one million new cases reported annually [2]. Brazil has about 90% of global cases each year, predominantly in rural areas with a high prevalence of *Leishmania amazonensis* as the etiological agent [5,6].

The treatment of leishmaniasis, including HTL, is a multifaceted issue that involves various factors, including the clinical status of the host, the high cost of available drugs, patient adherence, and the sensitivity and genetic variability of different *Leishmania* species [7]. Chemotherapeutic drugs licensed for the treatment of these parasites are limited in number and often cause severe adverse reactions, including death [8]. Furthermore, the emergence of drug-resistant strains poses an additional challenge [9]. The current treatment for cutaneous leishmaniasis includes the use of pentavalent antimonials and amphotericin B. These drugs are administered through injections or infusions and require medical supervision due to the risk of severe adverse effects [10]. Miltefosine has also shown promising results in the treatment of cutaneous leishmaniasis. It is an oral medication with a short treatment course, making it more accessible and convenient for patients. However, miltefosine is teratogenic and exhibits variable efficacy against different dermotropic *Leishmania* species [11]. These challenges emphasize the need for alternative therapies that are safer, more effective, and more accessible to patients.

*Allium sativum*, commonly known as garlic, is a plant that belongs to the Amaryllidaceae family. Its distinctive flavor and preservative effect make it a widely used food ingredient. Additionally, it has been traditionally used in medicine by ancient populations to combat various ailments, including skin diseases, intestinal parasites, and fever, as well as an antiseptic agent [12]. However, numerous studies have demonstrated its pharmacological potential, as previously reviewed [13,14]. Garlic extracts (aqueous and methanolic) and garlic-isolated compounds (allicin and ajoene) have shown promising results in treating leishmaniasis in vitro and in vivo [15]. Garlic essential oil, which is a complex mixture of volatile compounds, primarily allicin-derived allyl sulfides, also displays medicinal and therapeutic properties [16,17]. To date, the antileishmanial activity of *A. sativum* essential oil (ASEO) has not been reported, which motivated us to evaluate its effects against the dermotropic species *L. amazonensis.*

Here, we demonstrated that ASEO displays strong antileishmanial activity in vitro and interesting selectivity. Furthermore, computational predictions of the pharmacological and toxicological features of the major components of ASEO provide further evidence supporting the use of this oil as an alternative strategy to combat HTL.

## 2. Materials and Methods

### 2.1. Chemical Reagents and Culture Media

Fetal bovine serum (FBS) was purchased from LGC Biotecnologia (Cotia, SP, Brazil). The Schneider’s Insect Medium (SIM), Dulbecco’s Modified Eagle Medium (DMEM), and Grace’s Insect Medium (GIM), amphotericin B solution (AmB, 250 µg/mL), penicillin-streptomycin solution (5000 units penicillin and 5 mg streptomycin/mL), carbonyl cyanide 4-(trifluoromethoxy)phenylhydrazone (FCCP), thiazolyl blue tetrazolium Bromide (MTT), 2,2′ azobis (2-methylpropionamidine) dihydrochloride (AAPH), 2′,7′-dichlorofluorescin diacetate (H2DCFDA), lipopolysaccharide (LPS), and monodansylcadaverine (MDC) were purchased from Sigma-Aldrich (St. Louis, MO, USA). Dimethylsulfoxide (DMSO) was purchased from Synth (Diadema, SP, Brazil). A Panoptic staining kit was obtained from Laborclin (Pinhais, PR, Brazil). All other reagents were analytical grade.

### 2.2. Parasites and Cell Cultures

*Leishmania amazonensis* promastigotes, IFLA/BR/1967/PH8 strain, were cultured in SIM medium supplemented with 10% FBS at 26 °C. Axenic amastigotes were differentiated from late log-phase promastigotes using acidified GIM medium (10% FBS, pH 5.3) and an elevated temperature of 32 °C, as previously described [18]. The transformation from promastigotes to amastigotes was observed using light microscopy after a 96 h incubation period.

RAW 264.7 (murine macrophages), hFB (human fibroblasts), and VERO (primate kidney epithelial cells) cell lines were cultured in DMEM medium with 10% FBS and 1% penicillin-streptomycin solution (5000 units penicillin and 5 mg streptomycin/mL) at 37 °C in a 5% CO_2_ atmosphere. Cells were cultured for 48–72 h or until they reached sub-confluence.

Female BALB/c mice aged 6–8 weeks received 0.5 mL of a 3% thioglycollate solution by peritoneal injection. After 96 h, the animals were euthanized in accordance with institutional policies (ethical approval, 122/19-CEUA/UFRJ), and peritoneal lavage was performed using 10 mL of cold phosphate-buffered saline (PBS, pH 7.2). The thioglycollate-activated macrophages were then centrifuged at 2500 rpm for 10 min and resuspended in DMEM medium supplemented with 10% FBS and 1% penicillin-streptomycin solution at a 10^6^ cells/mL final density. These cells were used in both cytotoxic and anti-intracellular amastigote assays, as described below.

### 2.3. ASEO Phytochemical Analysis

The garlic essential oil used in this study was provided by Chr Hansen Industria e Comercio LTDA (Valinhos, SP, Brazil; lot SAFZ7M). The oil was obtained through a hydrodistillation process. Its chemical composition was determined using gas chromatography coupled to mass spectrometry (GC-MS) on an Agilent 6890N GC with a quadrupole mass spectrometer (Agilent 5973N) equipped with electronic impact ionization (70 eV). A DBS-5 column (30 m × 0.25 mm I.D., 0.25 μm film thickness) was used, and the injected volume was 1 μL in splitless mode. The injector temperature was set to 240 °C, the ion source was at 210 °C, and the scan range was 40–700 Daltons. The oven temperature ranged from 40 °C to 300 °C at a rate of 3 °C/min. Helium was used as the carrier gas with a flow rate of 0.5 mL/min. The results were expressed as the relative percentage of peak area in the chromatogram, and the interpretation and identification of the fragmentation mass spectra were performed by comparing them to the Wiley NBS mass spectrum database.

### 2.4. Viability Assay for Parasites

Promastigotes in the late log phase of growth and axenic amastigotes (10^6^ parasites/mL) were treated with various concentrations of ASEO (0.78–12.5 μg/mL) in SIM or GIM media, respectively, for 48 h in 96-well microplates. Untreated parasites were used as positive controls for viability, and AmB was used as the reference drug (0.12–10 μg/mL). The final concentration of DMSO in all treatment and control systems was maintained at 0.5%. Subsequently, promastigotes and axenic amastigotes were incubated at 26 °C and 32 °C, respectively. After the incubation period, parasite viability was spectrophotometrically determined (Spectramax i3x, Molecular Devices, San Jose, CA, USA) using the resazurin reduction method at 490/595 nm [19]. Furthermore, inhibited promastigote cultures were harvested and subsequently washed with PBS for drug removal. These cultures were then reintroduced into fresh SIM medium supplemented with FBS to assess whether the treatment had a leishmanistatic or leishmanicidal effect. The experiment was independently conducted in triplicate, with each replicate being repeated three times. The 50% inhibitory concentration (IC_50_) was calculated by performing nonlinear regression analysis of the ASEO dose-response curves.

### 2.5. Transmission Electron Microscopy (TEM)

Promastigotes and axenic amastigotes (10^7^ parasites/mL) were treated with their respective IC_50_ and 2xIC_50_ values for 48 h at 28 °C and 32 °C, respectively. After treatment, the parasites were washed twice with PBS and suspended in a 2.5% glutaraldehyde solution in sodium cacodylate buffer (0.1 M). Post-fixation was done with a 1% osmium tetroxide and 0.8% potassium ferrocyanide solution in a cacodylate buffer containing 3.7% sucrose and 5 mM calcium chloride for 1 h at room temperature. The parasites were then washed with cacodylate buffer, dehydrated in a gradient of acetone:epoxy resin (EPON) ranging from 3:7 to 0:10, and incubated at 68 °C for 72 h. Ultrathin sections were obtained using an ultramicrotome (Leica, Nussloch, Germany) and contrasted with a 5% uranyl acetate and 2% lead citrate solution. Finally, ultrastructural changes in parasites treated with ASEO were analyzed using an FEI Morgagni transmission electron microscope operating at 80 kV (FEI Company, Hillsboro, OR, USA).

### 2.6. Viability Assay for Mammalian Cells and Selectivity

The cytotoxic potential of ASEO was tested on VERO, RAW 264.7, hFB, and peritoneal macrophages (MØ) obtained as described above. Cellular suspensions were prepared at a concentration of 10^6^ cells/mL in fresh DMEM medium, and 100 µL of each suspension was distributed into 96-well microplates. The cultures were incubated at 37 °C and 5% CO_2_ atmosphere for 1 h to promote cell adhesion. The cells were rinsed twice with pH 7.2 PBS and then incubated for 48 h in the presence of varying concentrations of ASEO (7.8–1000 µg/mL) or AmB (3.1–25 µg/mL). Cell cultures added with fresh culture medium served as the positive control for assessing cell viability. Next, the MTT viability assay was performed as previously described [20]. The viable cells formed intracellular formazan crystals which were dissolved by adding 100 µL of DMSO to the cultures. Then, the absorbance was measured at 570 nm using a Spectramax^®^ i3x microplate reader (Molecular Devices, San Jose, CA, USA). The experiment was independently conducted in triplicate, with each replicate being repeated three times. The concentration of ASEO or AmB that caused a 50% decrease in cell viability (CC_50_) was calculated using nonlinear regression analysis of the dose-response curve. The selectivity index (SI), which reflects the relative toxicity of the compound to cells versus its efficacy against the target organism, was determined by dividing the CC_50_ by the concentration that reduced parasite viability by 50% (IC_50_).

### 2.7. Hemolytic Assay

To evaluate the hemolytic potential of ASEO, red blood cells (RBC) from *Ovis aries* blood samples were collected by centrifugation (2500 rpm/5 min) and then washed four times with cold PBS. A 4% (*v*/*v*) RBC suspension was prepared in PBS, and 80 µL of the suspension was transferred to 96-well microplates. Aliquots of ASEO (20 µL) at concentrations ranging from 7.8 to 2000 µg/mL were added to the microplates. The RBC were then incubated at 37 °C for 1 h. Hemolytic activity was terminated by adding 200 µL of PBS. Positive control of hemolysis (100%) was prepared by adding 200 µL of distilled water to untreated cells. The microplates were then centrifuged at 2500 rpm for 10 min, and the supernatant absorbance was measured at 540 nm using a Spectramax^®^ i3x microplate reader (Molecular Devices, San Jose, CA, USA). The experiment was independently conducted in triplicate, with each replicate being repeated three times. The hemolytic activity was calculated as follows: Hemolysis (%) = Abs_t_ × 100/Abs_cnt_, where Abs_t_: absorbance of treated cell supernatants; Abs_cnt_: absorbance of positive control of hemolysis [21].

### 2.8. Infection of Primary Macrophages and Anti-Intracellular Amastigote Assay

Primary macrophages were obtained as described above. Aliquots (100 µL) were taken from the cell suspension and distributed into 24-well microplates containing sterilized glass coverslips. After a 1-h incubation period at 37 °C and 5% CO_2_, stationary phase promastigote forms were added at a ratio of 10 parasites per macrophage. The macrophage-parasite interaction lasted for 16 h, and free parasites were removed by washing with PBS. Infected macrophages were subsequently treated with ASEO at concentrations below the CC_50_ value (0.93–15 µg/mL) for 48 h. Controls, consisting of untreated cultures or AmB-treated cultures, were also included. At the end of the treatments, culture supernatants were collected to measure NO production via the Griess reaction as described below. The cells were then fixed and stained with a Panoptic staining kit. The parasite load was determined by directly counting intracellular amastigotes in 100 macrophages [22], and the infection index was calculated as the product of the number of intracellular parasites and the percentage of infected macrophages [23]. The experiment was independently conducted in duplicate, with each replicate being repeated three times. In addition, the 50% inhibitory concentration was determined by performing nonlinear regression analysis on the ASEO dose-response curves.

### 2.9. Determination of Nitric Oxide

The Griess reaction [24] was performed to determine the NO production by infected MØ after treatment with ASEO, as described in Section 2.8. Briefly, 50 µL of the collected supernatants were distributed into 96-well microplates. Then, an equal volume of Griess reagent (0.5% sulfanilamide and 0.05% naphthylenediamine-bihydrochloride in 5% phosphoric acid) was added to each well. The plates were incubated at room temperature for 20 min in the dark. After incubation, the absorbance was measured at 570 nm using a Spectramax^®^ i3x microplate reader (Molecular Devices, San Jose, CA, USA). The experiment was independently conducted in duplicate, with each replicate being repeated three times. The nitrite concentrations were calculated using a sodium nitrite standard curve (0.195 to 100 µM).

The effect of ASEO on NO production by noninfected RAW 264.7 macrophages was also determined. The cells were obtained as described in Section 2.6 and treated with concentrations below the CC50 value (15.6–62.1 µg/mL) for 48 h at 37 °C and 5% CO_2_ atmosphere. Nontreated and LPS-activated macrophages (1 µg/mL) served as controls. After the treatment period, the culture supernatants were collected for the determination of NO, following the procedure described above. The experiment was independently conducted in triplicate, with each replicate being repeated three times.

### 2.10. Mitochondrial Membrane Potential (ΔΨm)

Axenic amastigotes of *L. amazonensis* (10^7^ parasites/mL) were treated with ASEO at IC_50_ and 2xIC_50_ for 4 h at 32 °C. After treatment, the parasites were washed with PBS and incubated with a 0.5 µg/mL rhodamine 123 solution for 20 min at 32 °C in the dark. Fluorescence was measured at 485/528 nm (excitation/emission) using a Spectramax^®^ i3x microplate reader. Controls included untreated parasites and parasites treated with 2 μM FCCP as a positive control for depolarization [25]. The experiment was independently conducted in triplicate, with each replicate being repeated three times.

### 2.11. Determination of Intracellular Reactive Oxygen Species (ROS)

Axenic amastigotes were treated with ASEO as described above. Following the treatment, the parasites were washed twice with PBS and their ROS levels were evaluated by adding 20 μM H2DCFDA. H2DCFDA is a non-fluorescent compound that becomes oxidized to dichlorofluorescein (DCF) in the presence of ROS. The resulting fluorescent compound was measured at 488/530 nm (excitation/emission). AAPH (1 mM) was used as a positive control for ROS production, while untreated amastigotes were used as a negative control [25,26]. The experiment was independently conducted in triplicate, with each replicate being repeated three times.

### 2.12. Autophagic Activity

Axenic amastigotes were treated with ASEO as described above. After the treatment period, the parasites were washed twice with PBS, and autophagic activity was evaluated by adding the MDC probe at 100 μM for 1 h at 32 °C in the dark [27]. Subsequently, the parasite culture was washed with PBS and fixed with 2% formaldehyde for 20 min at room temperature. MDC fluorescence was measured at 335/460 nm (excitation/emission). MDC is a fluorescent probe commonly used to label autophagic structures, due to its specificity for the accumulation in autolysosomes. Untreated amastigotes were used as a control for normal parasites. The experiment was independently conducted in triplicate, with each replicate being repeated three times.

### 2.13. In Silico Analysis

The chemical structures of ASEO’s major components, 1,2,4,6-tetrathiepane and diallyl disulfide, along with reference drugs, were obtained from the PubChem database (https://pubchem.ncbi.nlm.nih.gov/, accessed on 8 December 2022) in SDF format. In silico ADMET (absorption, distribution, metabolism, excretion, and toxicity) analysis was performed using the PreADMET web server (https://preadmet.bmdrc.kr/, accessed on 8 December 2022) to evaluate the following pharmacokinetic features: oral bioavailability (Lipinski’s “rule of five”), solubility, human intestinal absorption, intestinal and kidney cell permeability, plasma protein binding, CYP inhibition and substrate, blood-brain barrier penetration, and skin permeability. The toxicological features analyzed included mutagenicity (Ames test), carcinogenicity, and inhibition of human Ether-a-go-go-related genes (hERG). To compare pharmacokinetic parameters based on an experimental oral administration model, miltefosine was selected.

### 2.14. Statistical Analyses

The Shapiro–Wilk normality test was performed to evaluate the distribution of data. All data displayed normal distribution (*p* > 0.05). One-Way ANOVA, accompanied by Tukey’s post hoc test, was conducted using GraphPad Prism 8 (GraphPad Software, Boston, MA, USA). A significance level was adopted when *p* < 0.05.

## 3. Results and Discussion

Organosulfur compounds are the primary constituents of *A. sativum*-derived extracts, and they are responsible for most of the biological activities reported previously [13]. As expected, GC-MS analysis showed that the ASEO profile consisted mainly of organosulfur compounds (Table 1). The predominant component identified was 1,2,4,6-tetrathiepane (24.8%), followed by diallyl disulfide (16.75%). These findings are consistent with previous studies that reported the presence of allicin-derived sulfides [28,29]. However, this study is the first to report 1,2,4,6-tetrathiepane as a major component of garlic oil. This cyclic polysulfide has previously been found in *Lentinula edodes* (shiitake mushrooms) [30] and *Parkia speciosa* (bitter bean). Although there are few reports on the biological activity of 1,2,4,6-tetrathiepane, Morita and Kobayashi (1967) described its antimicrobial activity at concentrations ranging from 0.73 to 1.47 mM [31]. Diallyl disulfide has demonstrated hepatoregenerative effects by protecting against oxidative stress in the liver [32]. It has also been shown to have antioxidant activity by scavenging hydroxyl radicals and preventing lipid peroxidation [33]. Additionally, it has been found to prevent hepatic steatosis caused by early inflammation or ethanol [34] and non-alcoholic steatohepatitis by suppressing regulators of lipid metabolism and lipid peroxidation [35]. Its antioxidant and anti-inflammatory activity are attributed to the activation of erythroid-related nuclear factor 2 (Nrf2) [36]. Alnomasy (2021) demonstrated the efficacy of garlic oil, rich in diallyl disulfide and diallyl trisulfide, against the protozoan *Toxoplasma gondii* [37]. However, there is a lack of studies reporting on the activity of *A. sativum* essential oil (ASEO) or its major components, 1,2,4,6-tetrathiepane and diallyl disulfide, against *Leishmania* spp.

ASEO demonstrated strong antileishmanial activity against both promastigote and amastigote forms (axenic and intracellular) of *L. amazonensis*. The parasite inhibition curves are demonstrated in the supplementary material (Figure S1). The oil exhibited IC_50_ values ranging from 1.76 to 3.77 µg/mL (Table 2). Although promastigotes were more sensitive to the effects of ASEO (IC_50_ = 1.76 µg/mL), there was no significant difference observed among the developmental forms (*p* > 0.05). These results suggest that all developmental forms have similar susceptibility to ASEO. Since promastigotes can grow under culture conditions, we utilized this form to assess whether ASEO acts as a leishmanistatic or leishmanicidal agent. After re-incubating the inhibited cultures in fresh SIM medium, it was possible to confirm a leishmanicidal effect in parasites treated with 12.5 and 25 µg/mL. Despite the limited research on the antileishmanial activity of ASEO, different garlic extracts have been described as promising drug candidates. In a previous study, the methanolic extract of *A. sativum* demonstrated greater efficacy in reducing the viability of both *L. major* and *L. donovani* when compared to the reference drugs amphotericin B and sodium stibogluconate [38]. Similarly, the methanolic and aqueous extracts of garlic exhibited IC_50_ values of 12.3 and 19.2 µg/mL, respectively, which were comparable to the effectiveness of meglumine antimoniate against *L. tropica* [39]. The dichloromethane extract of garlic, rich in allicin and ajoene, demonstrated an IC_50_ of 2.89 µg/mL against *L. tarentolae* [40]. In the current study, ASEO demonstrated comparable activity to AmB (*p* > 0.05) specifically against the axenic amastigote form. However, it is worth noting that the effective concentrations of the volatile oil align with those reported for extracts containing different chemical constituents.

**Figure 1:**
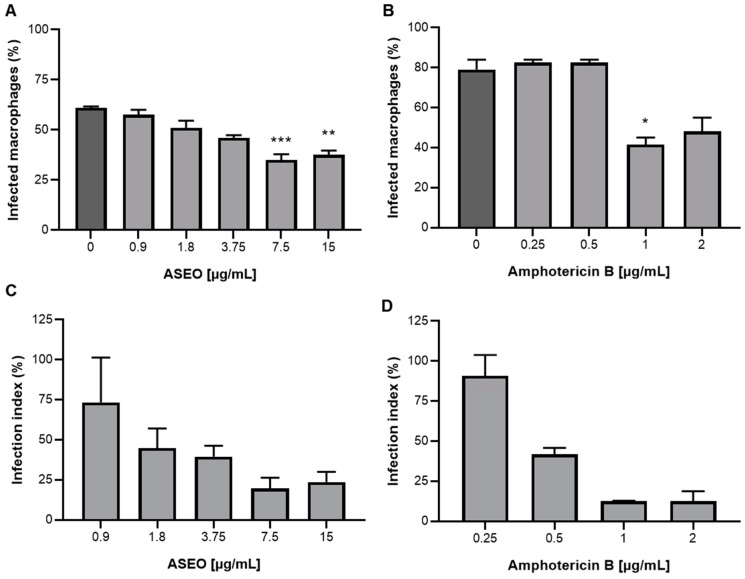
Effect of ASEO on in vitro treatment of primary macrophages infected with *L. amazonensis*. (**A**) Percentage of infected macrophages treated with ASEO; (**B**) Percentage of infected macrophages treated with amphotericin B; (**C**) Infection index of macrophages treated with ASEO; (**D**) Infection index of macrophages treated with amphotericin B. The bars in the graphs represent the mean values obtained from two independent experiments, with each experiment conducted in triplicate. Statistical analysis (**A**,**B**) was performed using one-way ANOVA followed by Tukey’s post-test, comparing each treatment group with the control. Significance levels are denoted as follows: * *p* < 0.05, ** *p* < 0.005, and *** *p* < 0.0005. The infection indexes were determined as a percentage relative to the control (untreated cultures). Therefore, no comparison was performed in this case, as the purpose was to assess the relative impact of the treatments compared to the untreated control.

The treatment of MØ with ASEO was effective in ameliorating the infection profile. Our results showed that the oil significantly decreased (*p* < 0.05) the number of infected host cells at 7.5 and 15 µg/mL (Figure 1A). Notably, the infection indexes of ASEO-treated cultures indicated a reduction (>50%) in parasite load at concentrations ≥1.8 µg/mL (Figure 1C). Indeed, garlic’s effect on macrophage infection by *Leishmania* spp. was previously described by other groups. Previously, it was demonstrated that the aqueous extract of garlic and its protein fraction enhanced the phagocytic activity and activation of macrophages, leading to a more effective response to *L. major* infection. Interestingly, the study suggests that mannose-binding lectins may play a role in facilitating interactions between the parasite and macrophages [41]. The aqueous extract of garlic also exhibits immunomodulatory effects by stimulating the production of IFN-y and NO in macrophages during *L. mexicana* infection [42].

**Figure 2:**
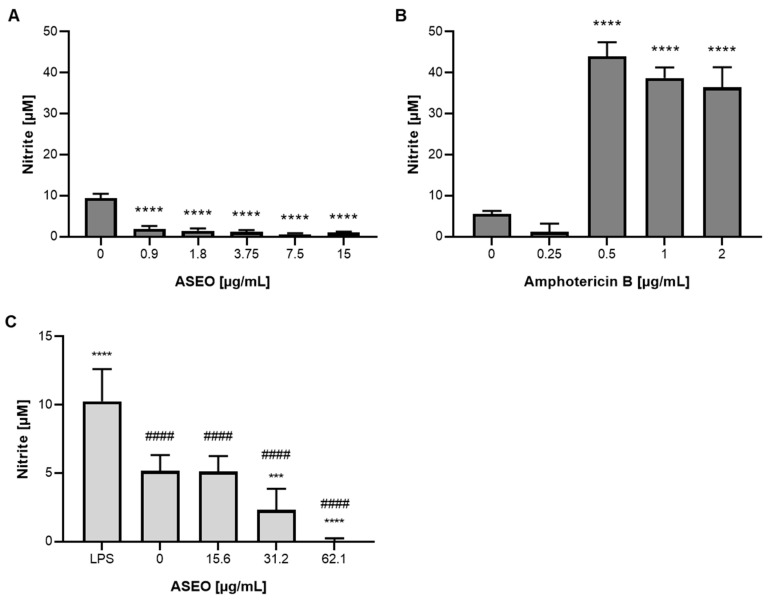
Effect of ASEO on NO production by macrophages. (**A**) *L. amazonensis*-infected MØ treated with ASEO; (**B**) *L. amazonensis*-infected MØ treated with amphotericin B; (**C**) Noninfected RAW 264.7 macrophages treated with ASEO. (**A**,**B**) The bars represent the mean values obtained from two independent experiments, with each experiment conducted in triplicate. (**C**) The bars represent the mean values obtained from three independent experiments, with each experiment conducted in triplicate. Statistical analysis was performed using one-way ANOVA followed by Tukey’s post-test, comparing each treatment group with the untreated cells (asterisks) or LPS (hashtags in (**C**)). Significance levels are denoted as follows: *** *p* < 0.0005, **** *p* < 0.0001; ^####^ *p* < 0.0001.

**Figure 3:**
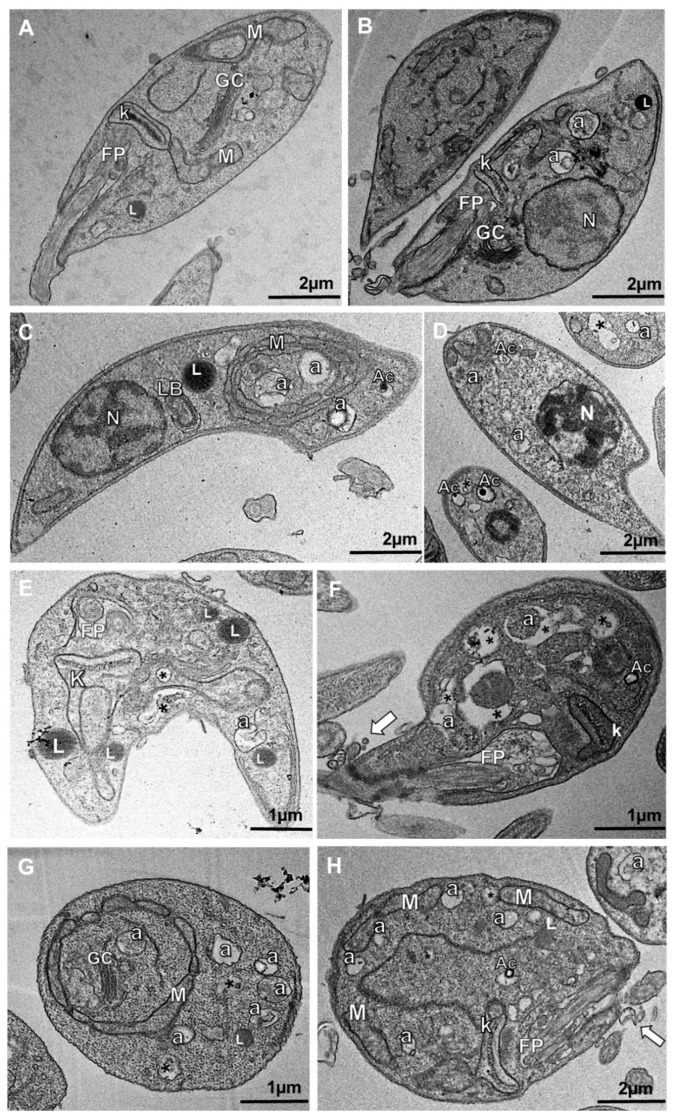
Photomicrographs of *L. amazonensis* promastigotes exposed to ASEO. (**A**,**B**) Control parasites displaying normal intracellular structures and elongated body shape; (**C**–**E**) Parasites treated with ASEO at IC_50_ concentration showing abnormal mitochondrial circular conformation (letter “M” in (**C**)), increased autophagic-like structures (letter “a” in (**C**)), lipid bodies (letter “L” in (**C**,**E**)), lamellar body (letters “LB” in (**C**)), and nuclear pyknosis (**D**); (**F**–**H**) Parasites treated with ASEO at 2xIC_50_ concentration showing similar alterations, including abnormal mitochondrial circular conformation (letter “M” in (**G**)), autophagic-like structures (letter “a” in (**F**–**G**)), and intense exocytosis (white arrow in (**F**,**H**)). M: mitochondrion; FP: flagellar pocket; k: kinetoplast; N: nucleus; Ac: acidocalcisomes; Asterisks (*): vacuoles.

The primary mechanism by which the host cell combats *Leishmania* spp. is through the generation of oxidative stress, which involves the increased production of reactive oxygen (ROS) and nitrogen (NO) species. To assess the impact of ASEO on the microbicidal mechanism in macrophages, which is crucial for disease control [43], we measured the levels of NO in the supernatant of *L. amazonensis*-infected MØ cultures (Figure 2). Surprisingly, although ASEO reduced infection, we observed a significant (*p* < 0.05) suppression of NO production by infected MØ (Figure 2A). In turn, the treatment of infected MØ with AmB at concentrations ≥0.5 µg/mL significantly increased (*p* > 0.05) NO levels (Figure 2B). In addition, the inhibitory effect of the oil on NO production was further confirmed in uninfected RAW264.7 macrophages (Figure 3C). A previous study has demonstrated that diallyl disulfide treatment inhibits NO production in RAW 264.7 macrophages, even in the presence of LPS stimulation [44,45]. In fact, NO-independent mechanisms of action have been proposed. Transchalcone has been found to effectively eliminate intracellular parasites of *L. amazonensis* independently of increased production of nitric oxide (NO) and ROS. Transchalcone upregulates the expression levels of Nrf2, a transcription factor that plays a role in altering the availability of iron for intracellular amastigotes [46]. Further assays are required to determine whether ASEO affects other microbicidal mechanisms employed by host macrophages.

Several studies have investigated the cytotoxicity of the *Allium* genus, especially against cancer cell lines [47]. In order to provide first insights into the safety of ASEO, cytotoxicity assays were conducted on a range of mammalian cells (Table 3). Allyl polysulfides (di-, tri-, and tetrasulfide) were identified as inducers of hemolytic anemia in a murine model. It is worth noting that allyl disulfide exhibited only a moderate effect [48]. However, in our current study, ASEO demonstrated no hemolytic effects, even at the highest concentration tested (2000 µg/mL). The lipophilic character of essential oils allows them to permeate and interact with plasma membranes. However, the specific chemical composition of essential oils plays a crucial role in determining their disruptive effects on cells, as previously demonstrated by Garcia et al. [18]. The absence of a hemolytic effect of ASEO may be related to the balance of its complex composition, which includes low amounts of diallyl trisulfide (1.4%) and diallyl disulfide (16.75%). The cytotoxicity of ASEO was not observed against VERO cells, which is a similar result to that of a garlic methanolic preparation previously described [38]. ASEO exhibited a moderate cytotoxic effect against human fibroblasts (hFB) with a CC_50_ value of 557.4 µg/mL. However, the oil displayed high SI values of 161 and 147.8 for axenic and intracellular amastigotes, respectively, indicating its potential for safe usage (Table 3). It is noteworthy that amphotericin B exhibited SI values of 63.88 and 110.7. Macrophages (*Leishmania* host cells) were found to be more sensitive to the cytotoxic effects of ASEO. The CC_50_ values determined for MØ and RAW 264.7 cells were 48.94 and 99 µg/mL, respectively. However, the oil demonstrated desirable SI values (>10) in accordance with the hit and lead criteria for drug discovery in the context of infectious diseases [49]. Furthermore, according to a screening algorithm developed by the Swiss Tropical Institute, which is recommended by the Drugs for Neglected Diseases initiative, crude extracts obtained from natural products are considered effective antileishmanial agents if they exhibit an IC_50_ below 20 µg/mL against *Leishmania* amastigote forms, along with a selectivity index (SI) for macrophages above 10 [50].

**Figure 4:**
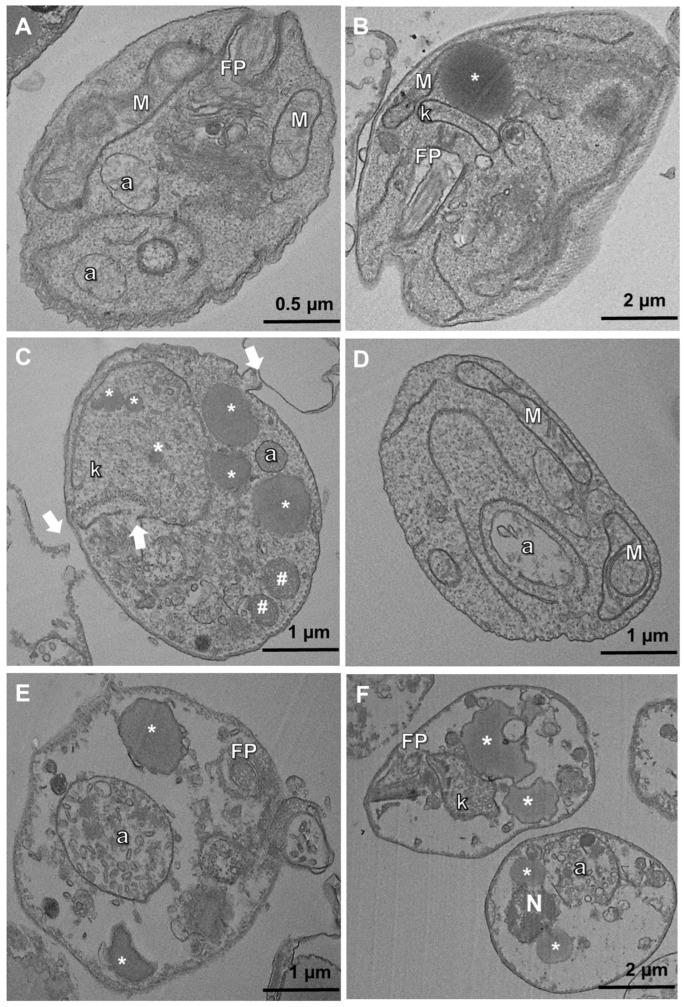
Photomicrographs of *L. amazonensis* axenic amastigotes exposed to ASEO. (**A**,**B**) Control parasites displaying normal intracellular structures and rounded body shape; (**C**,**D**) Parasites treated with ASEO at IC_50_ concentration showing mitochondrial swelling and increased number of lucent bodies (asterisks in (**C**)), plasmatic and mitochondrial membranes disruption (white arrows in (**C**)), enlarged autophagic-like structures (letter “a” in (**D**)); (**E**,**F**) Parasites treated with ASEO at 2xIC_50_ concentration showing sparse cytoplasm, enlarged autophagic-like structures (letter “a” in (**E**,**F**)) and lucent bodies (asterisks in (**E**,**F**)). M: mitochondria, FP: flagellar pocket, and k: kinetoplast; Asterisks (*): electron-lucent bodies; Hashtag (#): vacuoles.

A possible ASEO mode of action against *L. amazonensis* was investigated. Photomicrographs of promastigotes and axenic amastigotes treated for 24 h revealed important ultrastructural alterations (Figure 3 and Figure 4). The treatment of promastigotes with the IC_50_ concentration of ASEO resulted in notable ultrastructural alterations. These included the increased number of acidocalcisomes and the accumulation of lipid bodies and autophagic vacuoles (Figure 3C–E). Moreover, kinetoplast swelling (Figure 3E), nuclear pyknosis (Figure 3D), lamellar bodies, and abnormal circular conformation of mitochondria (Figure 3C) were also observed. After treatment with 2xIC_50_ of ASEO (Figure 3F–H), dilation of the flagellar pocket was observed, along with an increase in luminal exosomes containing cytoplasmic content (Figure 3F,H). This finding suggests that the observed increase in clearance activity may be an attempt at survival. Moreover, the accumulation of lipid bodies has been associated with alterations in sterol biosynthesis [51]. It is worth noting that the accumulation of lipids, along with the formation of autophagic vacuoles and lamellar bodies, has been reported as a consequence of sterol depletion and is considered a characteristic event of autophagic cell death [52].

**Figure 5:**
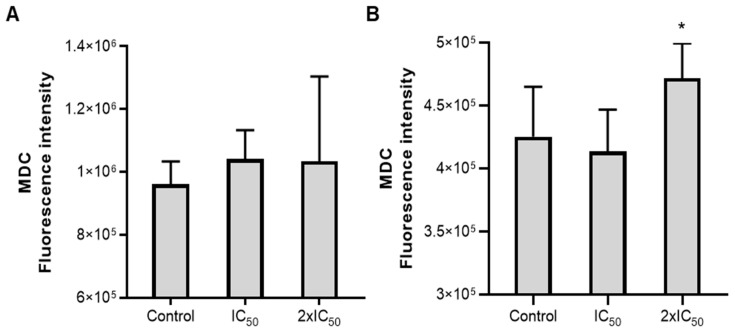
Effect of ASEO on the autophagic activity of *L. amazonensis* axenic amastigotes. Parasites were treated with ASEO at IC_50_ and 2xIC_50_ concentrations for 4 h (**A**) and 24 h (**B**). The bars represent the mean values obtained from three independent experiments, with each experiment conducted in triplicate. Statistical analysis was performed using one-way ANOVA followed by Bonferroni’s post-test, comparing each treatment group with the control. Significance levels are denoted as follows: * *p* < 0.05.

Regarding the effects of ASEO on axenic amastigotes, the main structural alterations observed included disruption of the plasma membrane, swelling of the kinetoplast (Figure 4C,F), sparse cytoplasm (Figure 4E,F), an increase in electron-lucent bodies, and the presence of several vacuoles indicating a vacuolization process along with the presence of autophagolysosomes (Figure 4C–F). The ability of essential oils to alter plasma membrane permeability can lead to membrane disruption and loss of cytoplasmic content, as previously demonstrated in the treatment of *L. amazonensis* with *Lippia sidoides* essential oil [53]. Interestingly, an increase in parasite membrane permeability was observed after treatment with ASEO, as indicated by the permeability assay using propidium iodide (data not shown). This finding supports the occurrence of damages to both the plasma membrane and cytoplasm. Previously, ajoene-treated (IC_50_ = 1.83 µM) promastigotes of *L. mexicana* showed autophagic vacuoles and loss of continuity of the plasmatic membrane [54]. The presence of autophagic vacuoles, lamellar bodies (myelin figures), mitochondrial swelling, and extensive cytoplasmic vacuolization were observed in parasites treated with essential oils from *Thymus capitellatus* [55], *Tetradenia riparia* [56], and *Cymbopogon citratus* [57]. These events indicate a significant remodeling process of intracellular organelles and suggest the occurrence of an autophagic process in response to the damage caused by the essential oils. Indeed, the enhancement of autophagic activity in ASEO-treated parasites was determined using MDC, a specific marker of autophagic vacuoles [58]. Following a 4 h treatment, an increase in autophagic activity was already evident in the parasites treated with ASEO at IC_50_ and 2xIC_50_ concentrations (Figure 5A). However, a significant increase (*p* < 0.05) was specifically observed after 24 h of exposure to the oil at the 2xIC_50_ value (Figure 5B). This result is consistent with the presence of autophagic structures observed in the photomicrographs (Figure 4).

**Figure 6:**
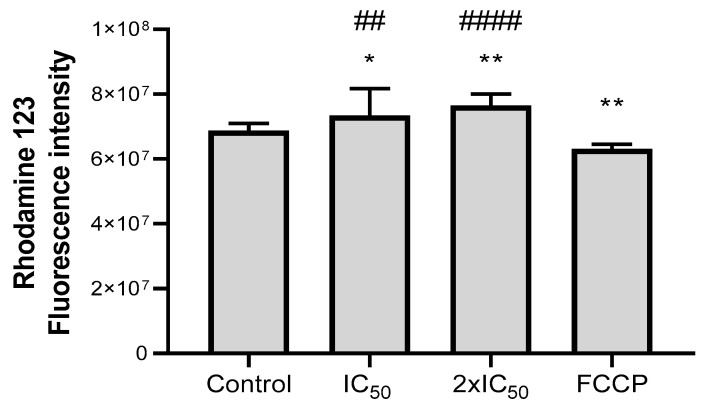
Effect of ASEO on mitochondrial membrane potential of *L. amazonensis* axenic amastigotes. Parasites were treated with ASEO at IC_50_ and 2xIC_50_ for 4 h. FCCP (2 µM) was used as a positive control of depolarization. The bars represent the mean values obtained from three independent experiments, with each experiment conducted in triplicate. Statistical analysis was performed using one-way ANOVA followed by Tukey’s post-test, comparing each treatment group (ASEO- or FCCP-treated parasites) with the control (asterisks). Hashtags indicate significant difference between ASEO-treated parasites and FCCP-treated parasites. Significance levels are denoted as follows: * *p* < 0.05; ** *p* < 0.005; ^##^ *p* < 0.01; and ^####^ *p* < 0.0001.

ASEO had a significant impact on the parasite mitochondria, as observed in Figure 3 and Figure 4. To further investigate this effect, the mitochondrial membrane potential of axenic amastigotes was assessed by exposing them to rhodamine 123 after treatment with ASEO. The treated parasites exhibited a higher intensity of fluorescence (indicating hyperpolarization) when compared to the control (untreated parasites) (Figure 6). This increase in fluorescence was statistically different (*p* < 0.05) from both the untreated and FCCP-treat parasites. The observed mitochondrial membrane hyperpolarization may occur as a response prior to depolarization, which is characteristic of apoptosis. It could be interpreted as the parasite’s last attempt to evade death [59]. In addition, mitochondrial membrane hyperpolarization has been associated with the deregulation of F0F1-ATPase [60] and an increased release of Ca^2+^ into the cytosol [61]. Corral et al. (2016) reported that allicin primarily targets the mitochondria of the parasite, leading to various events including elevated cytosolic Ca^2+^, generation of ROS, depolarization of mitochondrial membrane potential, and reduced ATP production [62]. In our study, allicin was not detected in garlic oil, suggesting that other organosulfur compounds present in the oil may have comparable effects on the mitochondrial membrane potential of parasites. It is worth mentioning that ASEO had no effect on intracellular ROS production by axenic amastigotes (Figure S2).

The safe usage of a new drug candidate can be predicted by computational tools. Here, we conducted ADMET analysis for the major components of ASEO, 1,2,4,6-tetrathiepane and diallyl disulfide, comparing them with the reference drug miltefosine (Table 4). Miltefosine was chosen since ADMET analysis is based on orally administered drugs. Both organosulfur compounds exhibit good oral bioavailability in accordance with Lipinski’s rule of 5. Based on the analysis of CYP450 enzymes (inhibition and substrate), the compounds derived from ASEO exhibit low hepatic metabolism. Notably, 1,2,4,6-tetrathiepane is a potential inhibitor of CYP3A4 and CYP2C19 isoforms. Inhibition of cytochrome P450 (CYP450) enzymes is a common mechanism leading to drug-drug interactions and hepatotoxicity [63]. Despite the low solubility, the organosulfur compounds showed good absorption and distribution parameters, such as a high rate of intestinal absorption and intestinal cell permeability (CACO2). Further, 1,2,4,6-Tetrathiepane exhibited comparable plasma protein binding rates and blood-brain barrier penetration to the reference drug miltefosine.

The computational ADMET analysis revealed that the main components of garlic oil, when assessed individually, were classified as mutagenic in the bacterial reverse mutation test (Ames’ test) and carcinogenic in murine models. Additionally, they exhibited a medium risk of hERG inhibition (Table 4). Despite that, our findings from different mammalian cell cytotoxic assays showed that ASEO exhibits promising selectivity against the parasite (SI > 10), irrespective of the cell type employed (whether cell lines or primary cells). It is important to note that organosulfur compounds, such as those found in garlic, are widely consumed and recognized for their functional properties, including cardioprotective and anti-inflammatory effects [64]. Considering these factors, ASEO holds promise as an anti-*Leishmania* agent. The computational data provided valuable insights into the pharmacological and toxicological characteristics of ASEO, as well as aiding in determining the optimal administration route for its use. Currently, in vivo studies are underway in our laboratory to further investigate the oil’s antileishmanial efficacy and toxicological features.

## 4. Conclusions

The findings reported here demonstrate the promising potential of ASEO and its organosulfur compounds as candidates for the treatment of leishmaniasis. ASEO exhibited significant inhibitory effects against *L. amazonensis* developmental forms at low concentrations. Moreover, it displayed low cytotoxicity on mammalian cells, and a selectivity more than 10 times higher for the parasite than for the host cells. Additionally, ASEO effectively reduced the parasite load in primary macrophages infected with *L. amazonensis*. ASEO did not induce oxidative stress in axenic amastigotes, despite targeting parasite mitochondria. Moreover, it increased the mitochondrial membrane potential and autophagic activity in the parasite. Additionally, the ADMET analysis revealed that ASEO’s main compounds, 1,2,4,6-tetrathiepane and diallyl disulfide, exhibit comparable pharmacokinetic and toxicological profiles to miltefosine. Therefore, ASEO is a promising candidate for the development of new strategies to treat HTL.

## Figures and Tables

**Table 1:** Chemical profile of *Allium sativum* essential oil.

Compound	RT (min)	Area (%)
Diallyl sulfide	10.103	4
N, N’-dimethylthiourea	14.434	8.7
Diallyl disulfide	19.909	16.75
Diallyl methyl trisulfide	22.353	3.61
1,3,5-Trithiane	23.148	8.7
Hexahydro-1,2,4,5-tetrazine-3,6-dithione	23.541	2.24
3-vinyl-1,2-dithiocyclohex-4-ene	24.455	1.2
1,3-Dithiolane-2-thione	24.93	7.15
Diallyl trisulfide	28.667	1.4
1,2,4,6-tetrathiepane	31.438	24.84
3,5-Diethyl-1,2,4-trithiolane	33.251	0.9
Isobutyl isothiocyanate	33.622	0.2
Propenylpropytrisulfide	33.875	2.4
2-(2-thia-4-pentenyl)-1-thia-cyclohex-5-ene	36.177	2
3H-1,2,4-Triazole-3-thione, 4,5-dihydro-4-methy	38.22	1.92
n-Butyl isothiocyanate	38.532	4.84
4,5-dimethyl thiazole	43.465	1.23
**Total**	-	92.08

**Table 2:** Half-maximal activity of ASEO against *L. amazonensis*. The experiments were performed independently in triplicate, with each replicate being repeated three times. The results are expressed as mean values µg/mL ± standard deviation.

Drugs	Promastigote	Amastigote^axe^	Amastigote^int^
ASEO	1.76 ± 0.37 ^a,A^	3.46 ± 0.43 ^a,A^	3.77 ± 0.74 ^a,A^
AmB	0.6 ± 0.01 ^a,B^	1.3 ± 0.5 ^a,A^	0.75 ± 0.03 ^a,B^

ASEO: *Allium sativum* essential oil; AmB: amphotericin B; axe: axenic amastigote; int: intracellular amastigote. The results are expressed as mean µg/mL ± standard deviation of three independent experiments performed in triplicate. Statistical analysis was performed using One-Way ANOVA with Tukey’s multiple comparisons test to determine significant differences (*p* < 0.05) among means within the same line, denoted by different lowercase letters. Student’s *t*-test was used to identify significant differences (*p* < 0.05) among means within the same column, indicated by different capital letters.

**Table 3:** Assessment of ASEO cytotoxicity and selectivity in different mammalian cells.

Mammalian Cells	ASEO			Amphotericin B		
	CC_50_ [µg/mL]	SI Ama^axe^	SI Ama^int^	CC_50_ [µg/mL]	SI Ama^axe^	SI Ama^int^
MØ	48.94 ± 3.78	14.14	13	18 ± 4.5	13.84	24
RAW 264.7	99 ± 9.7	28.6	26.25	n.d.	n.d.	n.d.
VERO	>2000	>500	>500	n.d.	n.d.	n.d.
hFB	557.4 ± 89.3	161	147.8	83.05 ± 0.65	63.88	110.7
RBC	>2000	>500	>500	n.d.	n.d.	n.d.

ASEO: *Allium sativum* essential oil; CC_50_: Cytotoxic concentration of 50% of cells; SI: Selectivity index (CC_50_/IC_50_); ama^axe^: axenic amastigote; ama^int^: intracellular amastigote; RBC: Red Blood Cells; MØ: peritoneal macrophages; n.d.: not determined.

**Table 4:** In silico ADMET data of 1,2,4,6-tetrathiepane and diallyl disulfide.

	1,2,4,6-Tetrathiepane	Diallyl Disulfide	Miltefosine
SP (logKp, cm/h)	−1.3	−1.15	−0.73
S (mg/L)	37.93	170.86	270.27
BS (mg/L)	0.001	24	25.41
PPB (%)	86.41	98	86.2
Pgp inhibition	non	inhibitor	inhibitor
MDCK (nm/s)	0.416	1.08	54.6
HIA (%)	98	98.17	98.35
CYP inhibition	3A4, 2C9, 2C19	2C9	2D6
CYP substrate	9A4 (weakly)	3A4, 2D6	3A4
ICP (nm/s)	56.24	22.01	21.7
BBB (brain/blood)	0.93	24.72	0.23
Rules of 5	suitable	suitable	suitable
Ames test	mutagen	mutagen	non
CarM	negative	positive	negative
CarR	positive	positive	positive
hERG inhibition	medium risk	medium risk	low risk

SP: Skin permeability (transdermal delivery), S: solubility in water, BS: Buffer solubility, PPB: Plasm Protein Binding, Pgp: P-glycoprotein inhibition, MDCK: In vitro Mandin Darby Canine Kidney Cell Permeability, HIA: Human intestinal absorption, CYP: cytochrome P5450 isoforms, ICP: Intestine Cell permeability (CACO2), BBB: Blood-Brain Barrier penetration, CarM: Carcinogenicity in Mouse, CarR: carcinogenicity in Rat, hERG: Inhibition of the human Ether-a-go-go Related Gene (potassium channel).

## Data Availability

The dataset analyzed during the current study is available from the last author on reasonable request.

## Supplementary Materials

The following supporting information can be downloaded at: https://www.mdpi.com/article/10.3390/tropicalmed8070375/s1, Figure S1: Antileishmanial activity of ASEO; Figure S2: Intracellular ROS production by *L. amazonensis* axenic amastigotes treated with ASEO.
