# Supplementary material for: Anti-Leishmania amazonensis Activity, Cytotoxic Features, and Chemical Profile of Allium sativum (Garlic) Essential Oil
Source: Trop Med Infect Dis. 2023 Jul 21;8(7):375. doi: 10.3390/tropicalmed8070375 (PMC10384145; doi:10.3390/tropicalmed8070375)
Supplement: Supplementary file 1 [file tropicalmed-08-00375-s001.zip › tropicalmed-2424429-supplementary.pdf]

Tropical Medicine and Infectious Disease

Supplementary material

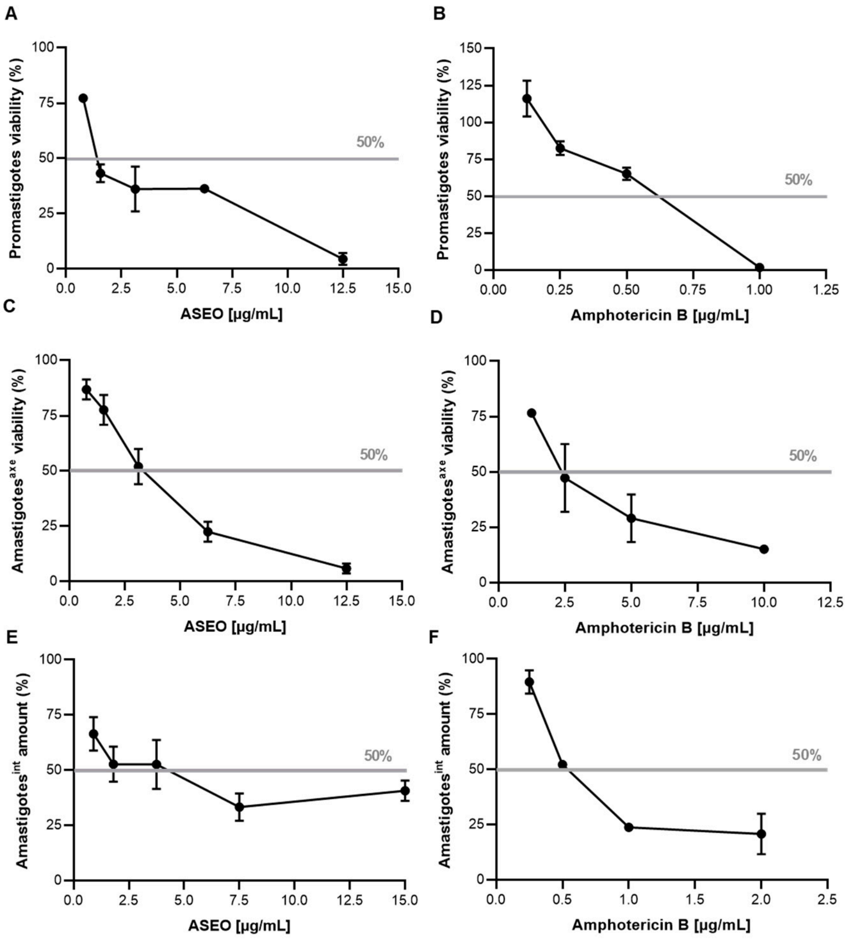

Figure S1. ASEO effect of ASEO on *Leishmania amazonensis* developmental forms. Parasite were treaed with several concentrations of ASEO for 48 h. Amphotericin B (AmB) was used as a positive control (reference drug). (A) ASEO-treated promastigotes; (B) AmB-treated promastigotes; (C) ASEO-treated axenic amastigotes; (D) AmB-treated axenic amastigotes; (E) ASEO-treated intracellular amastigotes; (F) AmB-treated intracellular amastigotes.

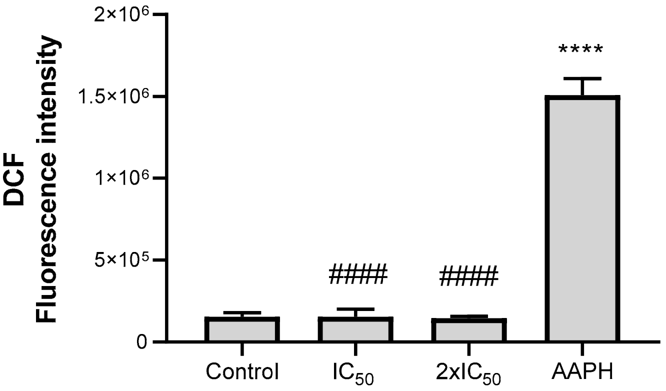

Figure S2. Intracellular ROS production by *L. amazonensis* axenic amastigotes treated with ASEO. Parasites were treated with ASEO at IC_{50} and $2xIC_{50}$ concentrations for 4 h. AAPH (1 mM) was used as a control for ROS production. Statistical analysis was performed using one-way ANOVA followed by Tukey's post-test, comparing each treatment with the controls. The hashtag symbols () indicate that the ASEO treatments were significantly different from the AAPH control (####$p < 0.0001$). The asterisks (****$p < 0.0001$) indicate a significant difference between the untreated and AAPH-treated parasites.
